# Effective Alternating Direction Optimization Methods for Sparsity-Constrained Blind Image Deblurring

**DOI:** 10.3390/s17010174

**Published:** 2017-01-18

**Authors:** Naixue Xiong, Ryan Wen Liu, Maohan Liang, Di Wu, Zhao Liu, Huisi Wu

**Affiliations:** 1 Hubei Key Laboratory of Inland Shipping Technology, School of Navigation, Wuhan University of Technology, Wuhan 430063, China; xiongnaixue@gmail.com (N.X.); mhliang@whut.edu.cn (M.L.); 2 Department of Business and Computer Science, Southwestern Oklahoma State University, Oklahoma, OK 73096, USA; 3 School of Computer, Wuhan University, Wuhan 430072, China; wudi2012@whu.edu.cn; 4 College of Computer Science and Software Engineering, Shenzhen University, Shenzhen 518060, China; hswu@szu.edu.cn

**Keywords:** imaging sensors, blind deblurring, image restoration, total variation, total generalized variation, alternating direction method of multipliers

## Abstract

Single-image blind deblurring for imaging sensors in the Internet of Things (IoT) is a challenging ill-conditioned inverse problem, which requires regularization techniques to stabilize the image restoration process. The purpose is to recover the underlying blur kernel and latent sharp image from only one blurred image. Under many degraded imaging conditions, the blur kernel could be considered not only spatially sparse, but also piecewise smooth with the support of a continuous curve. By taking advantage of the hybrid sparse properties of the blur kernel, a hybrid regularization method is proposed in this paper to robustly and accurately estimate the blur kernel. The effectiveness of the proposed blur kernel estimation method is enhanced by incorporating both the $L_{1}$-norm of kernel intensity and the squared $L_{2}$-norm of the intensity derivative. Once the accurate estimation of the blur kernel is obtained, the original blind deblurring can be simplified to the direct deconvolution of blurred images. To guarantee robust non-blind deconvolution, a variational image restoration model is presented based on the $L_{1}$-norm data-fidelity term and the total generalized variation (TGV) regularizer of second-order. All non-smooth optimization problems related to blur kernel estimation and non-blind deconvolution are effectively handled by using the alternating direction method of multipliers (ADMM)-based numerical methods. Comprehensive experiments on both synthetic and realistic datasets have been implemented to compare the proposed method with several state-of-the-art methods. The experimental comparisons have illustrated the satisfactory imaging performance of the proposed method in terms of quantitative and qualitative evaluations.

## 1. Introduction

### 1.1. Background and Related Work

**Figure 1:**
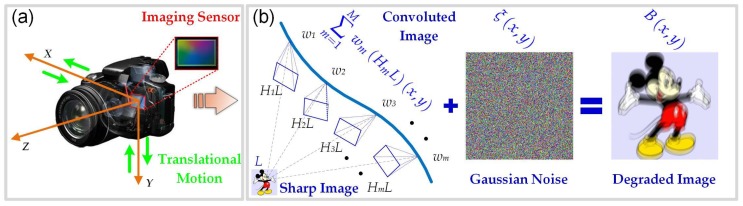
Diagram of the image degradation model for the motion blur of the imaging sensor in the Internet of Things (IoT). (**a**) Camera translation along both the *X* and *Y* axes considered in this work. (**b**) Discrete image degradation model with the curve being the sensor motion trajectory over the exposure time.

Single-image blind deblurring for imaging sensors has recently received increasing attention in modern imaging applications, e.g., the Internet of Things (IoT), astronomical imaging, biomedical imaging, computational photography and microscopy [1,2,3]. It is well known that the image pixel intensity can be determined by the total incoming light sensed by the imaging sensor over the exposure time. As shown in Figure 1, the discrete image degradation model can be written as follows:

(1) $$B\left(x,y\right)=\sum_{m=1}^{M}w_{m}\left(H_{m}L\right)\left(x,y\right)+\xi \left(x,y\right),$$

where $B\left(x,y\right)$ is the observed image after camera exposure, $L\left(x,y\right)$ denotes the latent sharp image to be restored, $\xi \left(x,y\right)$ denotes the additive white Gaussian noise, $w_{m}$ is a weight, which essentially represents the length of exposure time at camera pose *m*, and $H_{m}$ is a transformation matrix related to the camera rotation or translation at pose *m* during exposure. In this work, we only consider the case of uniform (i.e., spatially invariant) image deblurring. Thus, the matrix $H_{m}$ only corresponds to the camera translation along both the *X* and *Y* axes. For the sake of simplicity, the original image degradation model (1) can be rewritten as a convolution version as follows:

(2) B=L⊗k+ξ,

where ⊗ is the mathematical operation of convolution, *k* denotes the blur kernel related to the weight *w* and transformation matrix *H* in (1). The purpose of single-image blind deblurring is to recover both *k* and *L* from only one blurred image *B*. It is a challenging ill-conditioned inverse problem, since many different pairs *k* and *L* can lead to the same *B* [4]. The constraints on both the blur kernel and the latent sharp image should be exploited to select the optimal pairs *k* and *L* for enhancing imaging performance.

To cope with the ill-conditioned nature of blind deblurring, many statistical priors learned from blur kernels and latent sharp images have been developed to regularize the restoration process. In the literature [4,5], current single-image blind deblurring methods are widely divided into two categories: (1) methods that simultaneously estimate both the blur kernel and latent sharp image; (2) methods that first estimate the blur kernel, then recover the latent sharp image. It is well known that the support size of the blur kernel is often extremely smaller compared to the image size. Therefore, the joint maximum a posteriori (MAP) estimation of *k* and *L* often fails since the number of unknowns is larger than the number of known variables in *B*. In contrast, the estimation of the blur kernel can be obtained accurately through the MAP estimation of *k* alone [4]. To guarantee the high-quality blind deblurring, this paper mainly focuses on the second type of method, i.e., estimating the blur kernel first and then dealing with the corresponding non-blind deconvolution problem.

The pioneering work [6] mainly focused on the estimation of simple and small blur kernels, which are very rare in many practical scenarios. To make blind deblurring more practical, most current state-of-the-art methods were usually proposed by exploiting the prior knowledge from the statistics of blur kernels and sharp images. In 2006, Fergus et al. [7] contributed the original work on practical blind deblurring where the blur kernels were quite large and complex. In particular, the authors proposed a variational Bayesian image deblurring model by combining the mixture-of-Gaussian image prior with the mixture-of-exponential kernel prior. In [8], the blur kernel prior was assumed to follow an exponential distribution. Under the MAP framework, the exponential distribution results in an $L_{1}$-norm constraint on kernel intensity, which has a good interpretation on the sparsity of the blur kernel [9,10]. Under some imaging conditions, the blur kernel can also be assumed as a (piecewise) sufficiently smooth function. As a consequence, many researchers proposed to replace the $L_{1}$-norm of kernel intensity with its squared $L_{2}$-norm version [11,12,13]. Current experiments have shown that both $L_{1}$- and squared $L_{2}$-regularized optimization methods could achieve accurate kernel estimation on the benchmark dataset introduced by [4]. In the case of large blur kernels, however, it is difficult to robustly and accurately estimate the blur kernels using these methods mentioned. Many efforts [4,5] have been devoted to theoretically explain the reason why it is difficult for accurately estimating the blur kernels, especially for the large ones in practice. More recently, hybrid sparsity priors on blur kernels [14,15] have been considered and achieved robust image restoration results.

Once the blur kernel is estimated, the blind deblurring problem (2) essentially becomes a non-blind image deconvolution. During the past several decades, numerous numerical methods have been developed to handle non-blind deconvolution. One of the most popular methods is the Tikhonov regularization [16,17], followed by its various extensions [18,19]. These methods can be easily implemented, but commonly generate over-smoothing effects on the restored images. Other widely-used methods, such as the Richardson–Lucy method [20] and Wiener filter [21], easily suffer from noise amplification and ringing-like artifacts. To overcome the undesirable artifacts, Yuan et al. [22] proposed to develop a progressive inter-scale and intra-scale image deconvolution approach based on the bilateral Richardson-Lucy method. Current research illustrates that images have the properties of sparse gradients. Many efforts [23,24,25] were made to enhance non-blind deconvolution by imposing the total variation (TV) regularizer. From a statistical point of view, the TV regularizer corresponds to an assumption of a Laplacian sparse prior on image gradients. Recently, the extended TV regularizers, such as non-convex TV (NCTV) [11,13,26] and higher-order TV (HOTV) [27,28], have been attracting increasing attention for improving non-blind deconvolution. Both TV and HOTV regularizers have also been combined to overcome the potential disadvantages existing in these two regularizers [29,30,31]. The newly-developed total generalized variation (TGV) regularizer, originally proposed by Bredies et al. [32] in 2010, achieved great success for the restoration of blurred images [33,34,35]. Motivated by the concepts of non-local means (NLM) and graph Laplacian, the non-local TV (NLTV) regularizer has significantly improved the deconvolution quality [36,37,38,39]. The NLTV-regularized variational models can guarantee the highest-quality deconvolution because they take full advantage of the high degree of geometrical self-similarity that is inherent in natural images.

### 1.2. Motivation and Contributions

In the current literature [7,8,9,10,11,12,13], most existing blur kernel estimation methods were proposed based on the assumptions that the blur kernel was spatially sparse or piecewise smooth within the support of a continuous curve. As a consequence, the proposed methods could not always guarantee high-accuracy estimation under certain degradation conditions. In recent years, more attention has been paid to the sparse image priors for improving the estimation accuracy. To further enhance the estimation quality, in our opinion, it is still necessary to investigate advanced sparsity constraints on the blur kernel. The robust estimation method will be proposed in this paper by taking into account the sparsity and smoothing properties of the blur kernel. In particular, the sparsity property is promoted using the $L_{1}$-norm of kernel intensity; the smoothing property is utilized through the introduction of the squared $L_{2}$-norm of the intensity derivative. By making full use of the advantages of both the $L_{1}$-norm and the squared $L_{2}$-norm on kernel prior representation, the proposed method could potentially generate satisfactory estimation under more different degradation conditions. Essentially, most of the previous works [8,9,11,12,13] on blur kernel estimation can be considered as a special case of our proposed hybrid regularization method. If we only use the $L_{1}$-norm term, it can take full advantage of the property of spatial sparsity, but the resulting estimated blur kernel easily suffers from the isolated points [15]. If we only use the squared $L_{2}$-norm term, the continuous and smoothing properties of blur kernels under certain imaging conditions could be well preserved. However, the potential spatial sparsity property may be ignored, leading to inaccurate estimation of the blur kernel. Therefore, to guarantee the accuracy of the estimated blur kernel, it is necessary to combine the $L_{1}$-norm of kernel intensity with the squared $L_{2}$-norm of the intensity derivative in our work. If images are degraded by Gaussian, average or pillbox (disc) blur kernels, which have the weak spatial sparsity properties, but high smoothing properties, the proposed hybrid regularization method could theoretically generate higher estimation accuracy compared with traditional single regularization methods. It is worth mentioning that the hybrid blur kernel prior proposed in this work is extremely different from the current hybrid versions [14,15]. Current work [13,40,41,42] has illustrated that the $L_{0}$ quasi-norm has a good natural interpretation of the sparsity property of the image gradient and benefits for image detail enhancement. In particular, it performs well in penalizing small gradient magnitudes and encouraging large ones to preserve fine details. To improve the accuracy of blur kernel estimation, the $L_{0}$ quasi-norm of the image gradient is also incorporated into our blur kernel estimation method. Owing to the non-convex nature of the $L_{0}$ quasi-norm and the non-smooth nature of the $L_{1}$-norm, the commonly-used numerical methods could not be effectively adopted to solve the blur kernel estimation problem. To guarantee a feasible solution, the resulting non-convex non-smooth optimization problem will be effectively dealt with by developing an alternating direction method of multipliers (ADMM)-based numerical method [43]. The preliminary results on blur kernel estimation can be found in our previous short-version conference paper [44].

Existing work has illustrated that the TV regularizer, first proposed by Rudin et al. [23] in 1992, has the capacity of preserving edges and smoothing flat regions. TV-regularized variational image restoration models with the $L_{1}$-norm [11] or the squared $L_{2}$-norm [24] data-fidelity terms have gained considerable attention. However, the image quality could be degraded because the results often suffer from undesirable staircase-like artifacts in regions with gradual intensity variations [45]. The reason behind this phenomenon is that the TV regularizer favors solutions that are piecewise constant. To effectively suppress the artifacts, many extensions of TV [11,13,26,27,28,36,37,38,39] could be used to improve the image quality. For example, the patch-based NLTV regularizer has the capacity of guaranteeing the highest-quality image restoration. However, the NLTV-regularized variational model is practically limited due to the high computational cost. To make it easier to implement blind deblurring in practice, it is necessary to balance the trade-off between computational cost and imaging performance. Motivated by the success of the TGV regularizer, we tend to propose an effective non-blind deconvolution method based on the TGV regularizer of second-order (i.e., TGV${}^{2}$) [32]. The TGV${}^{2}$ regularizer is able to suppress the undesirable artifacts, while preserving the image edges since it favors piecewise polynomial intensities [34]. The quality of restored images could be correspondingly enhanced. From an optimization point of view, the resulting image deconvolution model could not be directly solved using traditional numerical methods because of the non-smooth nature of the TGV${}^{2}$ regularizer. To achieve a robust and effective solution, an ADMM-based optimization method will be developed to solve the resulting non-smooth minimization problem. In particular, the original complex minimization problem can be decomposed into several simple subproblems by introducing some auxiliary variables. Each of these subproblems has a closed-form solution or can be efficiently solved using the current numerical method. The effectiveness of the proposed method will be demonstrated using comprehensive experiments on both synthetic and realistic blurred images.

In conclusion, the main contributions of this paper, given the state-of-the-art research work, are mainly summarized by the following three aspects:

- To accurately estimate the blur kernel, a hybrid regularization method was proposed by combining the $L_{1}$-norm of kernel intensity with the squared $L_{2}$-norm of the intensity derivative. An alternating direction method was presented to effectively solve the resulting blur kernel estimation problem.
- The TGV${}^{2}$-regularized variational model with an $L_{1}$-norm data-fidelity term was proposed for enhancing the non-blind deconvolution result. To guarantee the stability and effectiveness of the solution, an ADMM-based numerical method was developed to solve the resulting non-smooth optimization problem.
- The satisfactory blind deblurring performance of the proposed method has been illustrated using comprehensive experiments on both synthetic and realistic blurred images (with large blur kernels). The proposed method has also been successfully exploited for single-image deblurring in the field of ocean engineering.

The main benefit of the proposed method is that it takes full advantage of the hybrid constraints for blur kernel estimation and the TGV${}^{2}$ regularizer for non-blind deconvolution. Therefore, it can accurately estimate the blur kernel and guarantee high-quality image deconvolution. Experiments using synthetic, as well as realistic blurred images will be implemented to verify the effectiveness of our proposed method in practical applications.

## 2. Hybrid Regularized Blur Kernel Estimation

**Figure 2:**
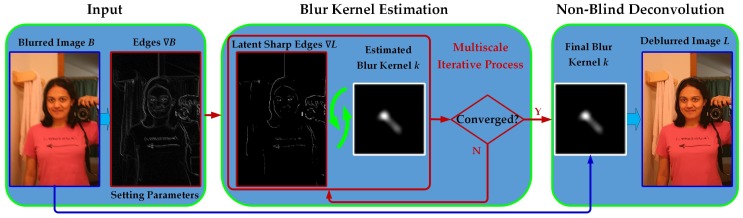
The illustration of our proposed robust regularization method for single-image blind deblurring. The proposed method first estimates the blur kernel, then recovers the latent sharp image.

As discussed in Section 1.2, our robust two-step framework for single-image blind deblurring is illustrated in Figure 2. This section mainly focuses on the first blur kernel estimation step, which is separated into the following two aspects: (1) sharp edges restoration; and (2) blur kernel estimation. In order to enhance the deblurring performance, we exploit the following statistical priors for blur kernel estimation: an $L_{0}$-sparsity prior on the latent gradient image *x* and a hybrid sparsity prior on the blur kernel *k*. Under these sparsity-constrained priors, blur kernel estimation in this paper is equivalent to solving the following minimization problem:

(3) $$\left(x^{*},k^{*}\right)=\underset{x,k}{\mathrm{argmin}}\left\{\frac{1}{2}{∥x⊗k-y∥}_{2}^{2}+\gamma {∥x∥}_{0}+\eta_{1}{∥k∥}_{1}+\eta_{2}{∥\nabla k∥}_{2}^{2}\right\},$$

where $\gamma ,\eta_{1},\eta_{2}$ are predefined positive regularization parameters, the $L_{0}$ quasi-norm ${∥\circ ∥}_{0}$ counts the number of nonzero elements, *x* denotes $\nabla L={\left(\partial_{h}L,\partial_{v}L\right)}^{T}$ and *y* denotes $\nabla B={\left(\partial_{h}B,\partial_{v}B\right)}^{T}$ with $\partial_{h}$ and $\partial_{v}$ being the finite differences along the horizontal and vertical directions, respectively. The proposed blur kernel estimation model (3) is mainly composed of four terms: The first term, called the squared $L_{2}$-norm data-fidelity term, denotes a measure of the distance between the restored data and the observed version. The second term is the $L_{0}$ quasi-norm regularization term, which can preserve the sparsity of natural image gradients. The third and fourth terms are, respectively, the $L_{1}$-norm and the squared $L_{2}$-norm constraints on the blur kernel, which can stabilize the final estimation result. The accurate estimation of the blur kernel is beneficial for generating high-quality non-blind image deconvolution.

### 2.1. Sharp Edge Restoration

Since *x* and *k* are independent in (3), in the first step, the latent sharp edges *x* at the (m+1)-th outer iteration can be recovered by solving the following minimization problem:

(4) $$x_{m+1}=\underset{x}{\mathrm{argmin}}\left\{\frac{1}{2}{∥x⊗k_{m}-y∥}_{2}^{2}+\gamma {∥x∥}_{0}\right\},$$

for $m=0,1,\cdots ,M_{\max}$ with $M_{\max}$ denoting the maximum number of outer iterations. As discussed in [11,13], strong edges are not always beneficial for accurate estimation of the blur kernel. To select the informative edges, an effective method used to measure the usefulness of gradients is given by:

(5) $$r\left(p\right)=\frac{{∥\sum_{q\in N_{h}\left(p\right)}\nabla B\left(q\right)∥}_{2}}{\sum_{q\in N_{h}\left(p\right)}{∥\nabla B(q)∥}_{2}+0.5},$$

where *B* is the blurred image and $N_{h}\left(p\right)$ denotes an h×h window centered at pixel p∈Ω (image domain). The measure metric (5), first proposed by Xu and Jia [11], enables accurate estimation of the blur kernel by removing some narrow strips. To incorporate the measure metric into our kernel estimation framework, the problem (4) can be reformulated as:

(6) $$x_{m+1}=\underset{x}{\mathrm{argmin}}\left\{\frac{1}{2}{∥K_{m}x-y∥}_{2}^{2}+\gamma {∥\kappa \circ x∥}_{0}\right\},$$

where ∘ represents the pointwise product and $\kappa \left(p\right)=\exp\left(-{\left|r\left(p\right)\right|}^{0.8}\right)$ for p∈Ω with the variable $r\left(p\right)$ being defined in (5). For the sake of simplicity, the convolution version in (6) is expressed in a matrix-vector multiplication form. In (6), $K_{m}$ is a block Toeplitz matrix with Toeplitz blocks transformed from the blur kernel *k* at the *m*-th outer iteration. x and y represent the vector version of *x* and *y*, respectively. It is well known that the model (6) is difficult to solve directly because of the non-smooth and non-convex natures of the $L_{0}$ quasi-norm ${∥\kappa \circ x∥}_{0}$. To guarantee solution efficiency and stability, we propose to develop an ADMM-based numerical method [43,46,47] to solve the unconstrained optimization problem (6). To apply ADMM, we first replace x by v and then transform (6) into the following constrained optimization problem:

(7) $$\min_{v,x}\left\{\frac{1}{2}{∥K_{m}x-y∥}_{2}^{2}+\gamma {∥\kappa \circ v∥}_{0}\right\},\;\;\;s.t.\;\;v=x.$$

Note that the updates of v and x are independent of each other. Let $L_{A}\left(v,x;\varphi^{x}\right)$ represent the augmented Lagrangian function of (7), which is defined as follows:

(8) $$L_{A}\left(v,x;\varphi^{x}\right)=\frac{1}{2}{∥K_{m}x-y∥}_{2}^{2}+\gamma {∥\kappa \circ v∥}_{0}+\frac{\beta_{1}}{2}{∥v-x∥}_{2}^{2}-\langle \varphi^{x},v-x\rangle ,$$

where $\beta_{1}$ is a pre-defined penalty parameter and $\varphi^{x}$ denotes the Lagrangian multiplier. In particular, ADMM solves problem (6) by minimizing $L_{A}\left(v,x;\varphi^{x}\right)$ with respect to v and x alternatively given the other fixed, followed by an update of the Lagrangian multiplier $\varphi^{x}$, i.e.,

(9) $$\left\{\begin{array}{c} \begin{array}{cc} v_{i+1} & =\underset{v}{\mathrm{argmin}}\left\{\gamma {∥\kappa \circ v∥}_{0}+\frac{\beta_{1}}{2}{∥v-\left(x_{m,i}+\frac{\varphi_{i}^{x}}{\beta_{1}}\right)∥}_{2}^{2}\right\}\\ x_{m,i+1} & =\underset{x}{\mathrm{argmin}}\left\{\frac{1}{2}{∥K_{m}x-y∥}_{2}^{2}+\frac{\beta_{1}}{2}{∥x-\left(v_{i+1}-\frac{\varphi_{i}^{x}}{\beta_{1}}\right)∥}_{2}^{2}\right\} \end{array} \end{array}\right.$$

with $x_{m,0}=x_{m}$. At each iteration, the Lagrangian multiplier $\varphi^{x}$ can be updated through $\varphi_{i+1}^{x}=\varphi_{i}^{x}-\tau \beta_{1}\left(v_{i+1}-x_{m,i+1}\right)$ for $i=0,1,\cdots ,I_{\max}$ with $I_{\max}$ denoting the maximum number of inner iterations. Here, $\tau \in \left(0,\left(\sqrt{5}+1\right)/2\right)$ denotes the step length. In order to accelerate the convergence of numerical solution, during each iteration, the penalty parameter $\beta_{1}$ can be updated as $\beta_{1}\leftarrow \rho \beta_{1}$ with *ρ* being a positive step length. The explicit solution of the v-subproblem in (9) can be directly obtained using the element-wise hard thresholding operator formulated in [48], i.e.,

(10) $$v_{i+1}=H_{\kappa \gamma ,\beta_{1}}\left(x_{m,i}+\frac{\varphi_{i}^{x}}{\beta_{1}}\right),$$

where $H_{a,b}\left(\cdot \right)$ is defined as:

$$\begin{array}{cc} H_{a,b}\left(s\right) & =\left\{\begin{array}{cc} 0, & \mathrm{if}\;\left|s\right|<\sqrt{2a/b},\\ s, & \mathrm{otherwise}. \end{array}\right. \end{array}$$

where both *a* and *b* are intermediate variables.

Essentially, the x-subproblem in (9) is a least-squares optimization problem; the corresponding normal equation can be readily obtained as follows:

(11) $$\left(K_{m}^{⊤}K_{m}+\beta_{1}I\right)x=K_{m}^{⊤}y+\beta_{1}\left(v_{i+1}-\frac{\varphi_{i}^{x}}{\beta_{1}}\right),$$

where superscript ⊤ denotes the transpose operator for real matrices or vectors and I denotes an identity matrix. Under the periodic boundary condition, $K_{m}^{⊤}K_{m}$ is a block circulant matrix with circulant blocks. It can be diagonalized using the two-dimensional discrete Fourier transform. Let F denote the forward fast Fourier transform (FFT) operator. Applying by F on both sides of (11) and yields:

(12) $$\left(\bar{F(K_{m})}F(K_{m})+\beta_{1}F\left(I\right)\right)F\left(x\right)=\bar{F(K_{m})}F\left(y\right)+\beta_{1}F\left(v_{i+1}-\frac{\varphi_{i}^{x}}{\beta_{1}}\right).$$

To decrease the computational cost, both $\bar{F(K_{m})}F(K_{m})+\beta_{1}F\left(I\right)$ and $\bar{F(K_{m})}F\left(y\right)$ can be computed only once at the beginning of the iterative algorithm. Thus, solving (12) is straightforward, which means that it is relatively easy to achieve the solution of (11). As a consequence, the solution of the least-squares optimization problem (11) is given by:

(13) $$x_{m,i+1}=F^{-1}\left(\frac{\bar{F(K_{m})}F\left(y\right)+\beta_{1}F\left(v_{i+1}-\varphi_{i}^{x}/\beta_{1}\right)}{\bar{F(K_{m})}F(K_{m})+\beta_{1}F\left(I\right)}\right),$$

where $F^{-1}\left(\cdot \right)$ denotes the inverse FFT operator and $\bar{F\left(\cdot \right)}$ represents the complex conjugate operator. The minimization process (9) is implemented alternately until the solution converges to the optimal one. Finally, the recovered sharp edge $x_{m+1}=x_{m,I_{\max}}$ is achieved to enhance the accuracy of blur kernel estimation in the next step. The whole optimization procedure of ADMM for Subproblem (6) is summarized in Algorithm 1.
**Algorithm 1** ADMM for Subproblem (6).1:**Input**: Blur kernel $k_{m}$, blurred image gradient y, τ=1.618, ρ=3, $I_{\max}=5$ and $\epsilon =5\times 10^{-6}$.2:**Initialize**: $\varphi_{0}^{x}=0$, $\beta_{1}=0.03$ and i=0.3:$x_{m,0}=x_{m}$.4:**while** (not converged and $i\leq I_{\max}$) **do**5:  Compute $v_{i+1}$ according to (10).6:  Compute $x_{m,i+1}$ according to (13).7:  Update Lagrangian multiplier and parameter: $\varphi_{i+1}^{x}=\varphi_{i}^{x}-\tau \beta_{1}\left(v_{i+1}-x_{m,i+1}\right)$ and $\beta_{1}\leftarrow \rho \beta_{1}$.8:  Check convergence condition: ${∥v_{i+1}-x_{m,i+1}∥}_{\infty }<\epsilon$9:**end while**10:$x_{m+1}=x_{m,I_{\max}}$.



### 2.2. Blur Kernel Estimation

In the blur kernel estimation step, given the recovered sharp edge $x_{m+1}$, the blur kernel k in (3) at the (m+1)-th outer iteration can be estimated by considering the following minimization problem:

(14) $$k_{m+1}=\underset{k}{\mathrm{argmin}}\left\{\frac{1}{2}{∥X_{m+1}k-y∥}_{2}^{2}+\eta_{1}{∥k∥}_{1}+\eta_{2}{∥\nabla k∥}_{2}^{2}\right\}.$$

where the optimal parameters $\eta_{1}$ and $\eta_{2}$ are manually selected by extensive experiments. It is obvious that Model (14) is essentially a convex optimization problem. Analogous to the optimization of problem (6), ADMM can also be adopted to efficiently solve (14) in our experiments. We first replace k by h and then obtain the corresponding augmented Lagrangian function $L_{A}\left(h,k;\varphi^{k}\right)$ as follows:

(15) $$L_{A}\left(h,k;\varphi^{k}\right)=\frac{1}{2}{∥X_{m+1}k-y∥}_{2}^{2}+\eta_{1}{∥h∥}_{1}+\eta_{2}{∥\nabla k∥}_{2}^{2}+\frac{\beta_{2}}{2}{∥h-k∥}_{2}^{2}-\langle \varphi^{k},h-k\rangle ,$$

where $\beta_{2}$ is a pre-defined penalty parameter and $\varphi^{k}$ denotes the Lagrangian multiplier. Given the fixed $k_{m,j}$, the minimization of $L_{A}\left(h,k;\varphi^{k}\right)$ with respect to h could be easily handled through the widely-used shrinkage operator [49,50,51], which operates pointwise on scalars or matrices. The solution $h_{j+1}$ at the (j+1)-th inner iteration is given by:

(16) $$\begin{array}{cc} h_{j+1} & =\underset{h}{\mathrm{argmin}}\left\{\eta_{1}{∥h∥}_{1}+\frac{\beta_{2}}{2}{∥h-\left(k_{m,j}+\frac{\varphi_{j}^{k}}{\beta_{2}}\right)∥}_{2}^{2}\right\}\\ & =\max\left\{\left|k_{m,j}+\frac{\varphi_{j}^{k}}{\beta_{2}}\right|-\frac{\eta_{1}}{\beta_{2}},0\right\}\circ \mathrm{sign}\left(k_{m,j}+\frac{\varphi_{j}^{k}}{\beta_{2}}\right), \end{array}$$

with $k_{m,0}=k_{m}$. Here, the sign function $\mathrm{sign}\left(\cdot \right)$ is defined as:

$$\begin{array}{cc} \mathrm{sign}\left(s\right) & =\left\{\begin{array}{cc} 1 & :s>0,\\ 0 & :s=0,\\ -1 & :s<0. \end{array}\right. \end{array}$$

Given the fixed $h_{j+1}$, the minimization of $L_{A}\left(h,k;\varphi^{k}\right)$ with respect to k is equivalent to solving a least-squares optimization problem. Analogous to solving the x-subproblem in (9), the corresponding solution $k_{m,j+1}$ is obtained as follows:

(17) $$k_{m,j+1}=F^{-1}\left(\frac{\bar{F(X_{m+1})}F(y)+\beta_{2}F\left(h_{j+1}-\varphi_{j}^{k}/\beta_{2}\right)}{2\eta_{2}\bar{F(\nabla )}F(\nabla )+\bar{F(X_{m+1})}F(X_{m+1})+\beta_{2}F\left(I\right)}\right).$$

It is tractable to obtain the efficient solution $k_{m,j+1}$ in (17) using one forward and one inverse FFT. At each iteration, the Lagrangian multiplier $\varphi^{k}$ can be updated as follows:

(18) $$\varphi_{j+1}^{k}=\varphi_{j}^{k}-\tau \beta_{2}\left(h_{j+1}-k_{m,j+1}\right),$$

for $j=0,1,\cdots ,J_{\max}$ with $J_{\max}$ denoting the maximum number of inner iterations. The estimated blur kernel $k_{m+1}=k_{m,J_{\max}}$ can be achieved for sharp edge restoration in the next step. Note that the convergence of our proposed numerical algorithm can be guaranteed according to the existing convergence results for ADMM in the literature [43,52,53]. Finally, our proposed hybrid regularized variational model for blur kernel estimation is summarized in Algorithm 2.
**Algorithm 2** Hybrid regularized blur kernel estimation.1:**Input**: Blurred image gradient y, τ=1.618, $\gamma =5\times 10^{-2}$, $\eta_{1}=\eta_{2}=10^{-3}$, and $M_{\max}=15$.2:**Initialize**: $k_{0}=uniform$ and m=0.3:**while** (not converged and $m\leq M_{\max}$) **do**// Step 1 : *Sharp Edges Restoration $x_{m+1}$*4: Update $x_{m+1}$ by Algorithm 1.// Step 2 : *Blur Kernel Estimation $k_{m+1}$*5: $k_{m,0}=k_{m}$.6: **for** j=0 to $J_{\max}$ **do**7:   Compute $h_{j+1}$ according to (16).8:   Compute $k_{m,j+1}$ according to (17).9:   Update Lagrangian multiplier: $\varphi_{j+1}^{k}=\varphi_{j}^{k}-\tau \beta_{2}\left(h_{j+1}-k_{m,j+1}\right)$.10: **end for**11: $k_{m+1}=k_{m,J_{\max}}$, γ←0.5γ.12:**end while**13:**Output**: Estimated blur kernel k.


## 3. Robust Non-Blind Deconvolution

This section mainly focuses on developing a high-order variational model for robust non-blind deconvolution. For the sake of better writing, the original image degradation model B=L⊗k+ξ in (2) can be rewritten as follows:

(19) B=KL+ξ.

Once the blur kernel K (i.e., k in Algorithm 2) is estimated accurately, blind deblurring can be simplified to the non-blind deconvolution problem. As a consequence, this problem could be handled through the commonly-used regularization methods. One of the most famous methods is the TV-regularized deconvolution method [23,24,25]. However, the undesirable staircase-like artifacts generated in restored images often lead to significant degradation of visual image quality. In order to enhance the imaging performance, a robust non-blind image deconvolution method will be proposed based on the second-order regularizer TGV${}^{2}$ [32]. Recently, TGV${}^{2}$ has been successfully utilized as a regularization scheme in various practical applications [33,54,55,56] and outperforms the popular TV regularizer. In particular, TGV${}^{2}$ is capable of preserving image edges and suppressing undesirable artifacts.

Inspired by the work in [11], the $L_{1}$-norm data-fidelity term will be incorporated into our TGV${}^{2}$-regularized non-blind deconvolution to suppress the potential outliers. The used assumption behind the squared $L_{2}$-norm data-fidelity term is that the data-fidelity costs follow a Gaussian distribution. This assumption often fails because the squared $L_{2}$-norm could make the restored images vulnerable to undesirable outliers. In contrast, the $L_{1}$-norm introduced in this work is much more robust to the presence of outliers compared with the squared $L_{2}$-norm. The proposed non-blind deconvolution model $L_{1}$-TGV${}^{2}$ is given by:

(20) $$L^{*}=\underset{L}{\mathrm{argmin}}\left\{{∥KL-B∥}_{1}+\lambda {\mathrm{TGV}}_{\alpha }^{2}\left(L\right)\right\},$$

where λ>0 denotes a predefined regularization parameter. For a scalar field $L\in L^{1}\left(\Omega \right)$, the discretized ${\mathrm{TGV}}_{\alpha }^{2}\left(L\right)$ [32] is defined as follows:

(21) $${\mathrm{TGV}}_{\alpha }^{2}\left(L\right)=\underset{V\in C_{c}^{2}\left(\Omega ,R^{2}\right)}{\mathrm{argmin}}\left\{\alpha_{1}{∥\nabla L-V∥}_{1}+\alpha_{0}{∥E\left(V\right)∥}_{1}\right\},$$

where $\alpha_{1}$ and $\alpha_{0}$ are positive tuning parameters, $C_{c}^{2}\left(\Omega ,R^{2}\right)$ denotes the space of the vector field and $E\left(V\right)=\frac{1}{2}\left(\nabla V+\nabla V^{T}\right)$ with $V={\left[V_{1}\;V_{2}\right]}^{T}$ being the symmetrized gradient of a complex-valued vector field V. Due to the non-smooth nature of the ${\mathrm{TGV}}^{2}$ regularizer, in this paper, we propose to develop an ADMM-based numerical algorithm to effectively solve the non-smooth optimization problem (20). Three auxiliary variables W, Y and Z are first introduced, and (20) is then transformed into the following constrained minimization problem:

(22) $$\begin{array}{c} \min_{W,Y,Z,L,V}\left\{{∥W∥}_{1}+\lambda \left(\alpha_{1}{∥Y∥}_{1}+\alpha_{0}{∥Z∥}_{1}\right)\right\}\\ \;\;\;\;\;\;s.t.\;\;\;\;W=KL-B,\;Y=\nabla L-V,\;Z=E(V). \end{array}$$

It is obvious that L and V are coupled together. For the fixed values of L and V, the updates of W, Y and Z are independent of each other. Thus, the variables W, Y, Z, L and V can be decomposed into two blocks, i.e., $\left(W,Y,Z\right)$ and $\left(L,V\right)$. Let $L_{A}\left(W,Y,Z,L,V;\xi ,\zeta ,\eta \right)$ denote the augmented Lagrangian function of (22), which can be defined as follows:

(23) $$\begin{array}{cc} L_{A}\left(W,Y,Z,L,V;\xi ,\zeta ,\eta \right) & ={∥W∥}_{1}+\frac{\rho_{1}}{2}{∥W-\left(KL-B\right)-\frac{\xi }{\rho_{1}}∥}_{2}^{2}\\ & +\lambda \alpha_{1}{∥Y∥}_{1}+\frac{\rho_{2}}{2}{∥Y-\left(\nabla L-V\right)-\frac{\zeta }{\rho_{2}}∥}_{2}^{2}+\lambda \alpha_{0}{∥Z∥}_{1}+\frac{\rho_{3}}{2}{∥Z-E\left(V\right)-\frac{\eta }{\rho_{3}}∥}_{2}^{2} \end{array}$$

where $\xi \in R^{mn}$, $\zeta \in R^{2mn}$ and $\eta \in R^{4mn}$ denote the Lagrange multipliers and $\rho_{1}$, $\rho_{2}$ and $\rho_{3}$ represent the positive penalty parameters that control the weights of penalty terms. It is numerically intractable to directly obtain the solutions of (22) through commonly-used methods [57]. In order to guarantee a stable solution, it is necessary to alternatively solve the W-, Y-, Z-, L- and V-subproblems and then update the Lagrange multipliers (i.e., *ξ*, *ζ* and *η*) until the obtained solution meets the predefined threshold. In particular, each of these subproblems has a closed-form solution or can be efficiently solved using the existing simple numerical method.

### 3.1. $\left\{W,Y,Z\right\}$-Subproblems

Given the fixed values of variables $L^{t}$, $V^{t}$, $\xi^{t}$, $\zeta^{t}$ and $\eta^{t}$, the W-subproblem, the Y-subproblem and the Z-subproblem in Equation (23) can be efficiently solved by considering the following $L_{1}$-regularized least-squares minimization problems:

(24) $$\begin{array}{cc} W^{t+1} & =\underset{W}{\mathrm{argmin}}\left\{{∥W∥}_{1}+\frac{\rho_{1}}{2}{∥W-\left(KL^{t}-B+\frac{\xi^{t}}{\rho_{1}}\right)∥}_{2}^{2}\right\}, \end{array}$$

(25) $$\begin{array}{cc} Y^{t+1} & =\underset{Y}{\mathrm{argmin}}\left\{\lambda \alpha_{1}{∥Y∥}_{1}+\frac{\rho_{2}}{2}{∥Y-\left(\nabla L^{t}-V^{t}+\frac{\zeta^{t}}{\rho_{2}}\right)∥}_{2}^{2}\right\}, \end{array}$$

(26) $$\begin{array}{cc} Z^{t+1} & =\underset{Z}{\mathrm{argmin}}\left\{\lambda \alpha_{0}{∥Z∥}_{1}+\frac{\rho_{3}}{2}{∥Z-\left(E\left(V^{t}\right)+\frac{\eta^{t}}{\rho_{3}}\right)∥}_{2}^{2}\right\}. \end{array}$$

Note that the unknown variables W, Y and Z are componentwise separable in the $\left\{W,Y,Z\right\}$-subproblems (24)–(26). These subproblem can be effectively dealt with through the commonly-used shrinkage operator [49,50]. This operator is fast and easy to implement in practice. The solutions $W^{t+1}$, $Y^{t+1}$ and $Z^{t+1}$ are obtained as follows:

(27) $$\begin{array}{cc} W^{t+1} & =\max\left\{\left|KL^{t}-B+\frac{\xi^{t}}{\rho_{1}}\right|-\frac{1}{\rho_{1}},0\right\}\circ \mathrm{sign}\left(KL^{t}-B+\frac{\xi^{t}}{\rho_{1}}\right), \end{array}$$

(28) $$\begin{array}{cc} Y^{t+1} & =\max\left\{\left|\nabla L^{t}-V^{t}+\frac{\zeta^{t}}{\rho_{2}}\right|-\frac{\lambda \alpha_{1}}{\rho_{2}},0\right\}\circ \mathrm{sign}\left(\nabla L^{t}-V^{t}+\frac{\zeta^{t}}{\rho_{2}}\right), \end{array}$$

(29) $$\begin{array}{cc} Z^{t+1} & =\max\left\{\left|E\left(V^{t}\right)+\frac{\eta^{t}}{\rho_{3}}\right|-\frac{\lambda \alpha_{0}}{\rho_{3}},0\right\}\circ \mathrm{sign}\left(E\left(V^{t}\right)+\frac{\eta^{t}}{\rho_{3}}\right), \end{array}$$

### 3.2. $\left(L,V\right)$-Subproblems

The minimization with respect to $\left(L,V\right)$ in (23) is essentially a least-squares optimization problem. However, it is impossible to directly obtain the solutions $L^{t+1}$ and $V^{t+1}$ through the forward and inverse FFT operators because the updates of L and V are coupled to each other. To guarantee the solution stability, the minimizations with respect to both $L^{t+1}$ and $V^{t+1}$ should be simultaneously implemented. Given the fixed values of $W^{t+1}$, $Y^{t+1}$, $Z^{t+1}$, $\xi^{t}$, $\zeta^{t}$ and $\eta^{t}$, the coupled $\left(L,V\right)$-subproblem in the augmented Lagrangian function (23) is quadratic, resulting in the following system of linear equations:

(30) $$\left\{\begin{array}{c} \begin{array}{cc} L^{t+1} & =\underset{L}{\mathrm{argmin}}\left\{\frac{\rho_{1}}{2}{∥KL-\left(W^{t+1}+B-\frac{\xi^{t}}{\rho_{1}}\right)∥}_{2}^{2}+\frac{\rho_{2}}{2}{∥\nabla L-\left(Y^{t+1}+V-\frac{\zeta^{t}}{\rho_{2}}\right)∥}_{2}^{2}\right\},\\ V^{t+1} & =\underset{V}{\mathrm{argmin}}\left\{\frac{\rho_{2}}{2}{∥V-\left(\nabla L-Y^{t+1}+\frac{\zeta^{t}}{\rho_{2}}\right)∥}_{2}^{2}+\frac{\rho_{3}}{2}{∥E\left(V\right)-\left(Z^{t+1}-\frac{\eta^{t}}{\rho_{3}}\right)∥}_{2}^{2}\right\}. \end{array} \end{array}\right.$$

Instead of directly solving the system of linear equations (30), we tend to solve the corresponding first-order necessary optimality conditions as follows:

(31) $$\left\{\begin{array}{c} \begin{array}{cc} & \left(\rho_{1}K^{⊤}K+\rho_{2}\nabla^{⊤}\nabla \right)L-\rho_{2}\partial_{x}^{⊤}V_{1}-\rho_{2}\partial_{y}^{⊤}V_{2}\\ & \;\;\;\;\;-\left[\rho_{1}K^{⊤}\left(W^{t+1}+B-\frac{\xi^{t}}{\rho_{1}}\right)+\rho_{2}\nabla^{⊤}\left(Y^{t+1}-\frac{\zeta^{t}}{\rho_{2}}\right)\right]=0, \end{array}\\ \begin{array}{cc} & -\rho_{2}\partial_{x}L+\left(\rho_{2}I+\rho_{3}\partial_{x}^{⊤}\partial_{x}+\frac{\rho_{3}}{2}\partial_{y}^{⊤}\partial_{y}\right)V_{1}+\frac{\rho_{3}}{2}\partial_{y}^{⊤}\partial_{x}V_{2}\\ & \;\;\;\;\;-\left[\rho_{2}\left(\frac{\zeta_{1}^{t}}{\rho_{2}}-Y_{1}^{t+1}\right)+\rho_{3}\left(\partial_{x}^{⊤}\left(Z_{1}^{t+1}-\frac{\eta_{1}^{t}}{\rho_{3}}\right)+\partial_{y}^{⊤}\left(Z_{3}^{t+1}-\frac{\eta_{3}^{t}}{\rho_{3}}\right)\right)\right]=0, \end{array}\\ \begin{array}{cc} & -\rho_{2}\partial_{y}L+\frac{\rho_{3}}{2}\partial_{x}^{⊤}\partial_{y}V_{1}+\left(\rho_{2}I+\rho_{3}\partial_{y}^{⊤}\partial_{y}+\frac{\rho_{3}}{2}\partial_{x}^{⊤}\partial_{x}\right)V_{2}\\ & \;\;\;\;\;-\left[\rho_{2}\left(\frac{\zeta_{2}^{t}}{\rho_{2}}-Y_{2}^{t+1}\right)+\rho_{3}\left(\partial_{y}^{⊤}\left(Z_{2}^{t+1}-\frac{\eta_{2}^{t}}{\rho_{3}}\right)+\partial_{x}^{⊤}\left(Z_{3}^{t+1}-\frac{\eta_{3}^{t}}{\rho_{3}}\right)\right)\right]=0. \end{array} \end{array}\right.$$

For the sake of better reading, the original system of linear equations (31) can be rewritten as follows:

(32) $$\left[\begin{array}{ccc} R_{1} & R_{4}^{⊤} & R_{5}^{⊤}\\ R_{4} & R_{2} & R_{6}^{⊤}\\ R_{5} & R_{6} & R_{3} \end{array}\right]\left[\begin{array}{c} L\\ V_{1}\\ V_{2} \end{array}\right]=\left[\begin{array}{c} D_{1}\\ D_{2}\\ D_{3} \end{array}\right],$$

with:

$$\left\{\begin{array}{c} \begin{array}{cc} & R_{1}=\rho_{1}K^{⊤}K+\rho_{2}\nabla^{⊤}\nabla ,\\ & R_{2}=\rho_{2}I+\rho_{3}\partial_{x}^{⊤}\partial_{x}+\frac{1}{2}\rho_{3}\partial_{y}^{⊤}\partial_{y},\\ & R_{3}=\rho_{2}I+\rho_{3}\partial_{y}^{⊤}\partial_{y}+\frac{1}{2}\rho_{3}\partial_{x}^{⊤}\partial_{x},\\ & \left(R_{4},R_{5},R_{6}\right)=\left(-\rho_{2}\partial_{x},-\rho_{2}\partial_{y},\frac{1}{2}\rho_{3}\partial_{x}^{⊤}\partial_{y}\right), \end{array} \end{array}\right.$$

and:

$$\left\{\begin{array}{c} \begin{array}{cc} D_{1} & =\rho_{1}K^{⊤}\left(W^{t+1}+B-\frac{\xi^{t}}{\rho_{1}}\right)+\rho_{2}\nabla^{⊤}\left(Y^{t+1}-\frac{\zeta^{t}}{\rho_{2}}\right),\\ D_{2} & =\rho_{2}\left(\frac{\zeta_{1}^{t}}{\rho_{2}}-Y_{1}^{t+1}\right)+\rho_{3}\left(\partial_{x}^{⊤}\left(Z_{1}^{t+1}-\frac{\eta_{1}^{t}}{\rho_{3}}\right)+\partial_{y}^{⊤}\left(Z_{3}^{t+1}-\frac{\eta_{3}^{t}}{\rho_{3}}\right)\right),\\ D_{3} & =\rho_{2}\left(\frac{\zeta_{2}^{t}}{\rho_{2}}-Y_{2}^{t+1}\right)+\rho_{3}\left(\partial_{y}^{⊤}\left(Z_{2}^{t+1}-\frac{\eta_{2}^{t}}{\rho_{3}}\right)+\partial_{x}^{⊤}\left(Z_{3}^{t+1}-\frac{\eta_{3}^{t}}{\rho_{3}}\right)\right). \end{array} \end{array}\right.$$

Let F denote the discrete Fourier transform operator for real (complex) matrices or vectors. To efficiently solve the system of linear equations (32), we multiply both sides of (32) by F, such that the coefficient matrix will be blockwise diagonal, i.e.,

(33) $$\left[\begin{array}{ccc} F\left(R_{1}\right) & F{\left(R_{4}\right)}^{⊤} & F{\left(R_{5}\right)}^{⊤}\\ F\left(R_{4}\right) & F\left(R_{2}\right) & F{\left(R_{6}\right)}^{⊤}\\ F\left(R_{5}\right) & F\left(R_{6}\right) & F\left(R_{3}\right) \end{array}\right]\left[\begin{array}{c} F\left(L\right)\\ F\left(V_{1}\right)\\ F\left(V_{2}\right) \end{array}\right]=\left[\begin{array}{c} F\left(D_{1}\right)\\ F\left(D_{2}\right)\\ F\left(D_{3}\right) \end{array}\right].$$

Essentially, (33) is a linear system with three equations and three variables. We propose to directly use Cramer’s rule to effectively yield the closed-form solutions as follows:

(34) $$L^{t+1}=F^{-1}\left(\frac{\det_{L}}{\det_{T}}\right),\;V_{1}^{t+1}=F^{-1}\left(\frac{\det_{V_{1}}}{\det_{T}}\right)\;\mathrm{and}\;V_{2}^{t+1}=F^{-1}\left(\frac{\det_{V_{2}}}{\det_{T}}\right),$$

where $F^{-1}\left(\circ \right)$ represents the inverse Fourier transform operator. In particular, we have the determinants $\det_{L}={\left|\begin{array}{ccc} S & T_{2} & T_{3} \end{array}\right|}_{*}$, $\det_{V_{1}}={\left|\begin{array}{ccc} T_{1} & S & T_{3} \end{array}\right|}_{*}$, $\det_{V_{2}}={\left|\begin{array}{ccc} T_{1} & T_{2} & S \end{array}\right|}_{*}$ and $\det_{T}={\left|\begin{array}{ccc} T_{1} & T_{2} & T_{3} \end{array}\right|}_{*}$ with $T_{1}={\left[F{\left(R_{1}\right)}^{⊤}\;F{\left(R_{4}\right)}^{⊤}\;F{\left(R_{5}\right)}^{⊤}\right]}^{⊤}$, $T_{2}={\left[F\left(R_{4}\right)\;F{\left(R_{2}\right)}^{⊤}\;F{\left(R_{6}\right)}^{⊤}\right]}^{⊤}$, $T_{3}\;=\;{\left[F\left(R_{5}\right)\;F\left(R_{6}\right)\;F{\left(R_{3}\right)}^{⊤}\right]}^{⊤}$ and $S={\left[F{\left(D_{1}\right)}^{⊤}\;F{\left(D_{2}\right)}^{⊤}\;F{\left(D_{3}\right)}^{⊤}\right]}^{⊤}$. The definition of determinant ${\left|\;\cdot \;\right|}_{*}$ in this work is briefly introduced as follows:

$$\begin{array}{c} {\left|\begin{array}{ccc} r_{11} & r_{12} & r_{13}\\ r_{21} & r_{22} & r_{23}\\ r_{31} & r_{32} & r_{33} \end{array}\right|}_{*}=\left[\begin{array}{c} \;\;r_{11}\circ r_{22}\circ r_{33}+r_{12}\circ r_{23}\circ r_{31}\\ +r_{13}\circ r_{21}\circ r_{32}-r_{13}\circ r_{22}\circ r_{31}\\ -r_{12}\circ r_{21}\circ r_{33}-r_{11}\circ r_{23}\circ r_{32} \end{array}\right]. \end{array}$$

To shorten the computational time, all matrix elements in $\det_{T}$ for (34) should be calculated before the execution of our ADMM-based numerical algorithm. At each iteration, $D_{1}$, $D_{2}$ and $D_{3}$ are first computed; $S={\left[F{\left(D_{1}\right)}^{⊤}\;F{\left(D_{2}\right)}^{⊤}\;F{\left(D_{3}\right)}^{⊤}\right]}^{⊤}$ can be easily calculated correspondingly. The final solutions $L^{t+1}$, $V_{1}^{t+1}$ and $V_{2}^{t+1}$ can be naturally obtained using Cramer’s rule (34).

### 3.3. Update the Lagrange Multipliers

At each iteration of our proposed numerical method, the Lagrange multipliers $\left(\xi ,\zeta ,\eta \right)$ should be updated as follows:

(35) $$\begin{array}{cc} \xi^{t+1} & =\xi^{t}-\tau \rho_{1}\left(W^{t+1}-\left(KL^{t+1}-B\right)\right), \end{array}$$

(36) $$\begin{array}{cc} \zeta^{t+1} & =\zeta^{t}-\tau \rho_{2}\left(Y^{t+1}-\left(\nabla L^{t+1}-V^{t+1}\right)\right), \end{array}$$

(37) $$\begin{array}{cc} \eta^{t+1} & =\eta^{t}-\tau \rho_{3}\left(Z^{t+1}-E\left(V^{t+1}\right)\right), \end{array}$$

where the step length τ=1.618 is used throughout this paper. In conclusion, an ADMM-based numerical method was proposed to decompose the original complex optimization problem (20) into several simpler subproblems. Each subproblem has a closed-form solution or can be efficiently solved using the existing numerical method. In particular, the $\left\{W,Y,Z\right\}$-subproblems (24–26) could be easily solved using the shrinkage operator. The solutions of L and V were simultaneously obtained through Cramer’s rule (34). The optimization procedure of our proposed method for non-blind image deconvolution is summarized in Algorithm 3.
**Algorithm 3** ADMM for the $L_{1}$-TGV${}^{2}$ Model (20).1:**Input**: Blurred image B, blur kernel *k* (i.e., K in Section 3), $\rho_{1}=50$, $\rho_{2}=0.5$, $\rho_{3}=5$, $\alpha_{1}=1$, $\alpha_{2}=1.5$, τ=1.618, $T_{\max}=10$ and $\epsilon =5\times 10^{-5}$.2:**Initialize**: $L^{0}=B$, $V^{0}=0$, $\xi^{0}=0$, $\zeta^{0}=0$, $\eta^{0}=0$ and t=0.3:**while** (not converged and $t\leq T_{\max}$) **do**4:  Compute $W^{t+1}$ according to $W^{t+1}=\mathrm{shrinkage}\left(KL^{t}-B+\xi^{t}/\rho_{1},1/\rho_{1}\right)$.5:  Compute $Y^{t+1}$ according to $Y^{t+1}=\mathrm{shrinkage}\left(\nabla L^{t}-V^{t}+\zeta^{t}/\rho_{2},\lambda \alpha_{1}/\rho_{2}\right)$.6:  Compute $Z^{t+1}$ according to $Z^{t+1}=\mathrm{shrinkage}\left(E\left(V^{t}\right)+\eta^{t}/\rho_{3},\lambda \alpha_{0}/\rho_{3}\right)$.7:  Compute $\left(L^{t+1},V^{t+1}\right)$ according to$\;\;\;\;\;\;\;\;L^{t+1}=F^{-1}\left(\frac{\det_{L}}{\det_{T}}\right),\;V_{1}^{t+1}=F^{-1}\left(\frac{\det_{V_{1}}}{\det_{T}}\right)\;\mathrm{and}\;V_{2}^{t+1}=F^{-1}\left(\frac{\det_{V_{2}}}{\det_{T}}\right).$8:  Update Lagrangian multipliers $\left(\xi ,\zeta ,\eta \right)$:$\;\;\;\;\;\;\;\;\xi^{t+1}=\xi^{t}-\tau \rho_{1}\left(W^{t+1}-\left(KL^{t+1}-B\right)\right).$$\;\;\;\;\;\;\;\;\zeta^{t+1}=\zeta^{t}-\tau \rho_{2}\left(Y^{t+1}-\left(\nabla L^{t+1}-V^{t+1}\right)\right).$$\;\;\;\;\;\;\;\;\eta^{t+1}=\eta^{t}-\tau \rho_{3}\left(Z^{t+1}-E\left(V^{t+1}\right)\right).$9:  Check convergence condition: ${∥L^{t+1}-L^{t}∥}_{\infty }<\epsilon$10:**end while**11:Output: Deblurred image L.



## 4. Experimental Results and Discussion

Comprehensive blind deblurring experiments on both synthetic and realistic blurred images will be performed to verify the effectiveness of our proposed method in this section.

### 4.1. Experimental Settings

The proposed blind deconvolution framework was evaluated on a synthetic blur-image dataset [4] and realistic blurred images. The synthetic dataset has been widely exploited as a benchmark dataset to evaluate the performance of blur kernel estimation. Our numerical experiments were implemented using MATLAB R2011a (The MathWorks, Natick, Inc., MA) on a machine with a 3.30-GHz Intel(R) Pentium(R) G3260 CPU and 4 GB RAM. For both synthetic and realistic datasets, the parameter values for blur kernel estimation in Section 2 were set as follows: τ=1.618, ρ=3, $I_{\max}=5$, $\gamma =5\times 10^{-2}$, $\eta_{1}=\eta_{2}=10^{-3}$ and $M_{\max}=15$. The resulting optimal parameters for non-blind deconvolution in Section 3 were set empirically, i.e., $\rho_{1}=50$, $\rho_{2}=0.5$, $\rho_{3}=5$, $\alpha_{1}=1$, $\alpha_{2}=1.5$ and $T_{\max}=10$. The deblurring results have illustrated the satisfactory performance of the manually-selected parameters in our experiments. For the sake of better comparison, the competing blind deblurring methods yield the restoration results with the input parameters manually optimized by the authors. To further improve deblurring performance, there is a great potential to develop automatic estimation methods to adaptively select the optimal parameters in our future work. Similar to [4], the sum of squared differences (SSD) and SSD ratio were used simultaneously to quantitatively evaluate the performance of blur kernel estimation in Section 4.2. In particular, the SSD ratio is measured between the deconvolution error with the estimated blur kernel and the deconvolution error with the ground truth kernel [4].

### 4.2. Experiments on Synthetically-Blurred Images

**Figure 3:**
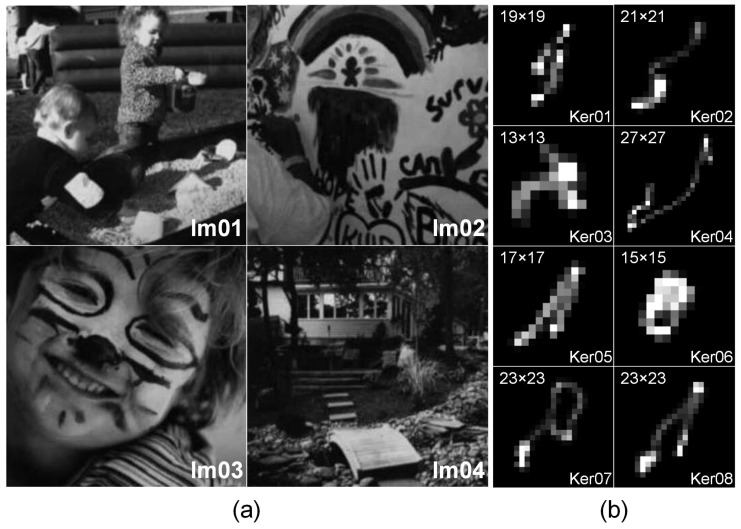
The experimental dataset of [4]. From left to right: (**a**) four gray-scale test images of the size 256×256 and (**b**) eight uniform blur kernels of different sizes (the blur kernel sizes are illustrated in the upper-left panels), resulting in 32 test images in our synthetic experiments.

**Figure 4:**
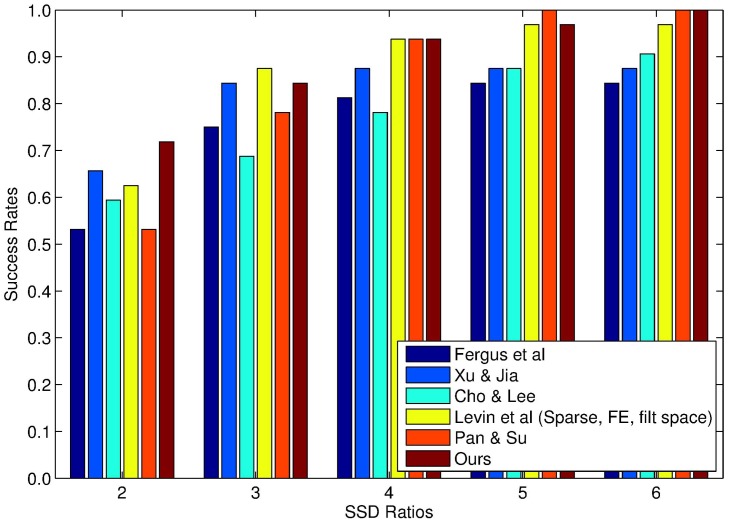
Cumulative histograms of the sum of squared differences (SSD) ratios on the blur-image dataset of Levin et al. [4].

Numerous experiments are implemented in this subsection to evaluate the performance of our proposed method on one widely-used synthetic blur image dataset [4], which can be downloaded from the link: www.wisdom.weizmann.ac.il/~levina/papers/LevinEtalCVPR2011Code.zip. In Figure 3, it could be found that the dataset is composed of four grayscale images of the size 256×256 and eight different uniform blur kernels, resulting in a total of 32 synthetic blurred images in our experiments. The proposed method will be compared with several state-of-the-art blind deblurring methods [7,11,12,13,58] in terms of SSD and the SSD ratio. In order to guarantee an unbiased comparison, the final deblurred results are all generated using the sparse non-blind deconvolution method proposed in [58]. Furthermore, to enhance the robustness of blur kernel estimation, a widely-used multiscale scheme [7] was introduced in Algorithm 2. Experimental results on the SSD ratio for different kernel estimation methods are summarized in Figure 4. It can be found that our proposed method is able to generate more robust estimation results on this synthetic dataset under consideration in most of the cases. In contrast, the accuracy of blur kernel estimation for other competing methods is limited due to the simple assumption of the blur kernel prior.

The objective function Equation (3), comprising the $L_{0}$ quasi-norm and the $L_{1}$-norm regularization terms, is both non-convex and non-smooth. Thus, it is much more difficult to estimate the complexity of the proposed method from a theoretical point of view. It is well known that computational cost is highly dependent on the algorithm complexity. For the sake of simplicity, we only tend to compare the computational time for different blur kernel estimation methods under the same imaging conditions. The methods of Xu and Jia [11] and Cho and Lee [12] are efficiently implemented using C code in our experiments. In contrast, the other competing methods are performed in MATLAB. Since all test images in Figure 3 are mainly composed of fine details of different textures, we take images Im02 and Im04 as examples to evaluate the computational efficiency. The computational time of different competing blur kernel estimation methods is summarized in Table 1. It could be found that the methods of Xu and Jia [11] and Cho and Lee [12] generate the lowest computational cost due to the C implementation. Our proposed method yields significantly faster computational speeds compared with Fergus et al. [7] and Levin et al. [58]. However, the method of Pan and Su [13] achieves the highest computational efficiency due to the fast alternating direction optimization method. As shown in Table 1, the proposed method yields the best evaluation results in terms of the SSD metric under consideration in most of the cases. The satisfactory performance of our proposed method benefits from the combination of the $L_{1}$-norm of kernel intensity with the squared $L_{2}$-norm of the intensity derivative. It could be further observed that both computational cost and image quality highly depend on the size of the blur kernel. As the size becomes larger, the computational cost obviously becomes higher, and the image quality could becomes lower for all competing methods under different imaging conditions. In addition, there is no significant difference in blind deblurring performance between different test images for the same blurring degradation.

**Figure 5:**
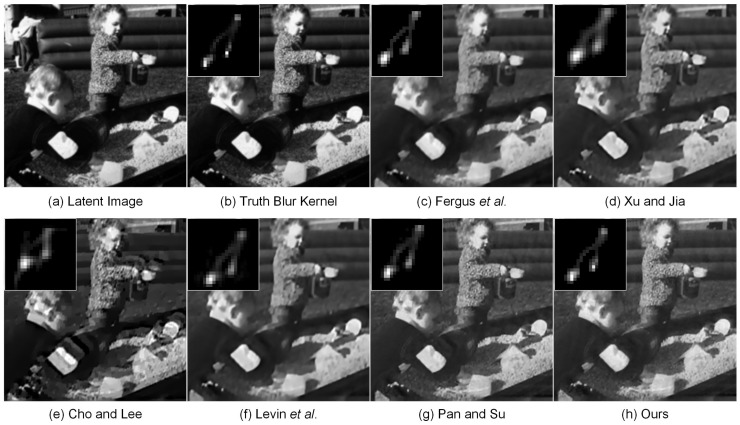
Comparison of results on one test image from [4]. From top-left to bottom-right: (**a**) latent sharp image, deblurred versions with (**b**) the truth blur kernel, estimated blur kernels generated by (**c**) Fergus et al. [7], (**d**) Xu and Jia [11], (**e**) Cho and Lee [12], (**f**) Levin et al. [58], (**g**) Pan and Su [13] and (**h**) our proposed method, respectively. The estimated blur kernels are illustrated in the upper-left panels.

For a better comparison, Figure 5 visually displays a test image from the synthetic blur-image dataset [4] recovered by different deblurring methods. As can be observed, our proposed method estimates a more accurate blur kernel. The proposed method generates the highest quality of deblurred image, since it achieves a more natural-looking appearance. We can conclude that our proposed method has superior performance compared with other competing methods.

### 4.3. Experiments on a Large Blur Kernel

Single-image blind deconvolution with a large blur kernel is an extremely challenging problem in practical application. It is necessary to investigate whether our proposed method is able to deal with the large blur kernel. In this subsection, our proposed method will be compared with three state-of-the-art deblurring methods, i.e., Xu and Jia [11] in ECCV-2010, Krishnan et al. [9] in CVPR-2011 and Pan et al. [59] in CVPR-2016. Once the blur kernel is estimated, the method of Xu and Jia [11] implements a robust non-blind deconvolution by integrating the $L_{1}$-norm data-fidelity term and the TV regularizer. Krishnan et al. [9] directly uses the hyper-Laplacian prior-based fast non-blind deconvolution method [26] to generate the final recovered image. The non-blind deconvolution results in our proposed method will be achieved using the combination of the $L_{1}$-norm data-fidelity term and the TGV${}^{2}$ regularizer summarized in Algorithm 3. In contrast, the method in Pan et al. [59] simultaneously estimates the latent sharp image and blur kernel by introducing the assumption of the dark channel prior.

**Figure 6:**
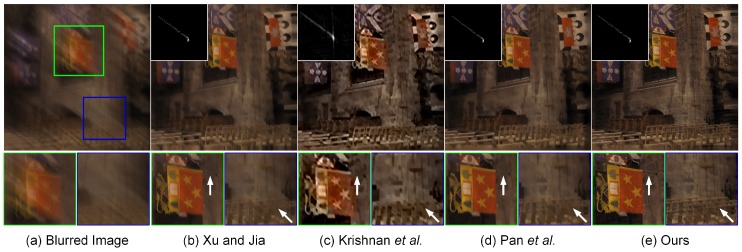
Restoration of a blurred image with a large motion kernel of the size 159×159. From left to right: (**a**) input blurred image, deblurred versions generated by (**b**) Xu and Jia [11], (**c**) Krishnan et al. [9], (**d**) Pan et al. [59] and (**e**) our proposed method, respectively. The estimated blur kernels and local magnification views respectively are illustrated in the upper-left and bottom panels.

**Figure 7:**
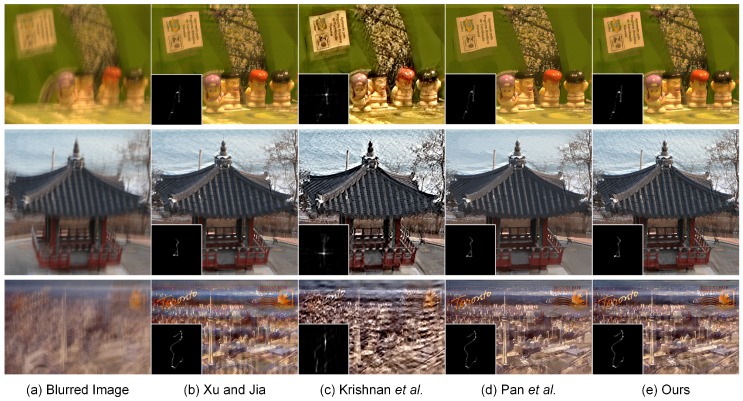
Blind deconvolution of three different realistic images. From left to right: (**a**) input blurred image, deblurred versions generated by (**b**) Xu and Jia [11], (**c**) Krishnan et al. [9], (**d**) Pan et al. [59] and (**e**) our proposed method, respectively. The sizes of the estimated blur kernels from top to bottom are 101×101, 95×95 and 91×91, respectively.

The blind deblurring results with large blur kernels are visually displayed in Figure 6 and Figure 7. As shown in Figure 6, the method of Krishnan et al. [9] is unable to yield the satisfactory estimation of the large blur kernel of the size 159×159. The resulting deblurred image suffers from the loss of important geometrical structures, which significantly degrades the visual quality. In contrast, the methods of Xu and Jia [11], Pan et al. [59] and our proposed method have the capacity of guaranteeing the accurate estimation of the blur kernel. The main geometrical structures in the deblurred images could be reconstructed correspondingly. However, an excess smoothing could be observed in the methods of Xu and Jia [11] and Pan et al. [59], which leads to the loss of small structural features. Owing to the second-order regularizer TGV${}^{2}$, our non-blind deconvolution method produces a much more natural-looking result than Xu and Jia [11] and Pan et al. [59]. As shown by the arrows in Figure 6, more details could be preserved in our proposed method; thus, the resulting deblurring performance outperforms other comparative methods. More blind deblurring results with large blur kernels are visually illustrated in Figure 7. The sizes of the estimated blur kernels in our experiments are 101×101, 95×95 and 91×91, respectively. It could be found that the method of Krishnan et al. [9] fails to yield the accurate estimation of the blur kernel and generates unsatisfactory deblurring performance. In contrast, the proposed method is able to generate comparable results to the state-of-the-art blind deblurring methods, i.e., Xu and Jia [11] and Pan et al. [59]. The final high-quality recovered images could be achieved with more structures and details preserved. Therefore, there is a huge potential to use our proposed method to restore the blurred images with large blur kernels in practice.

### 4.4. Experiments on Ocean Engineering

**Figure 8:**
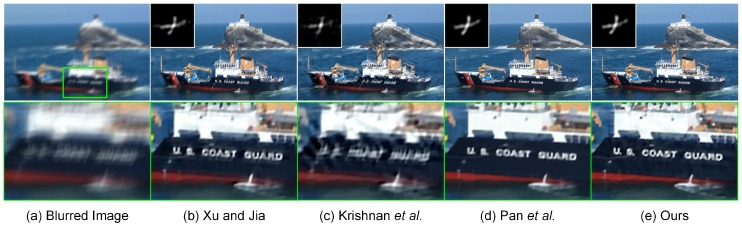
Restoration of a blurred image captured with an airborne camera. From left to right: (**a**) input blurred image, deblurred versions generated by (**b**) Xu and Jia [11], (**c**) Krishnan et al. [9], (**d**) Pan et al. [59] and (**e**) our proposed method, respectively. The estimated blur kernels of the size 35×35 and local magnification views respectively are illustrated in the upper-left and bottom panels.

**Figure 9:**
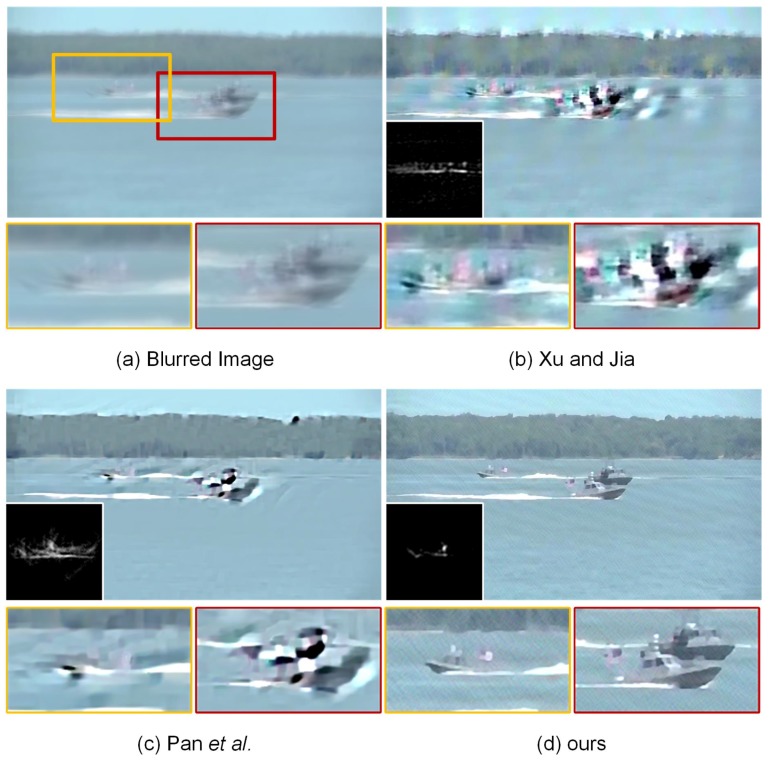
Restoration of a blurred image captured with a shipborne camera. From left to right: (**a**) input blurred image, deblurred versions generated by (**b**) Xu and Jia [11], (**c**) Pan et al. [59] and (**d**) our proposed method, respectively. The estimated blur kernels of the size 95×95 and local magnification views respectively are illustrated in the bottom-left and bottom panels.

In the field of ocean engineering, computer vision-assisted automatic detection and tracking systems with airborne and shipborne imaging sensors have been widely used to improve maritime control, safety and rescue operations. However, the resulting imaging performance sometimes suffers from motion blur, noise, haze and sensor nonlinearities, which could significantly degrade the visual image quality under poor weather conditions. In this paper, we mainly focus our attention on the restoration of blurred images, since this degradation condition is more common than other conditions in ocean engineering. The experimental images captured with airborne and shipborne imaging sensor systems, as well as the corresponding blur kernel estimation and image deconvolution results are visually displayed in Figure 8 and Figure 9. The sizes of the estimated blur kernels are 35×35 and 95×95, respectively. As shown in Figure 8, the method of Krishnan et al. [9] is unable to achieve high-quality blur kernel estimation resulting in unsatisfactory deblurring performance with ringing-like artifacts. In contrast, Pan et al. [59] and our proposed method have the capacity of producing accurate estimation of the blur kernel for this example. The final high-quality restored images could be guaranteed using non-blind deconvolution methods. However, due to the low-contrast structure shown in Figure 9, the latest dark channel prior-based method [59] could not accurately estimate the blur kernel in the case of shipborne imaging. The reason behind this phenomenon may be that the statistical properties between images captured by shipborne cameras and natural images are essentially different. The assumption of the dark channel prior is not always valid under different imaging conditions. The method of Xu and Jia [11] also fails to estimate the blur kernel and generates a low-quality restored image. Figure 9 visually illustrates that our proposed method is still able to guarantee accurate kernel estimation and image deconvolution. More geometric structures and fine details could be preserved in our recovered images, beneficial for detecting and tracking moving vessels in practice. The maritime control, safety and rescue operations could be correspondingly improved in the field of maritime management and ocean engineering.

### 4.5. Experiments on More Realistic Blurred Images

**Figure 10:**
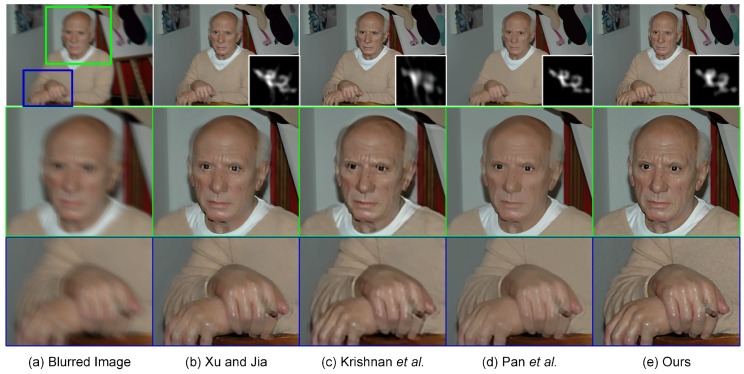
Single-image blind deblurring results with a blur kernel of size 27×27 (top) and their local magnification views (middle and bottom). From left to right: (**a**) input blurred images, deblurred versions generated by (**b**) Xu and Jia [11], (**c**) Krishnan et al. [9], (**d**) Pan et al. [59] and (**e**) our proposed method, respectively.

To better evaluate our proposed method, this subsection is concluded by testing blind deblurring on more realistic human and nature images. Our experimental results will be compared with the recovered results generated by the three above-mentioned methods, i.e., Xu and Jia [11], Krishnan et al. [9] and Pan et al. [59]. The estimation of the blur kernel for each method is implemented directly using the codes and parameter settings provided by the authors. As shown in Figure 10, the recovered result generated by Krishnan et al. [9] suffers from the over-smoothing of detailed texture structures due to the inaccurate estimation of the blur kernel. The loss of geometrical structures easily makes the deblurred image look less natural, resulting in significant visual quality degradation. The local magnification views shown in Figure 10 visually illustrate that the proposed method yields a more “natural-looking” performance compared with Xu and Jia [11] and Pan et al. [59]. In particular, more geometrical details in the face and hand regions could be preserved in our proposed method. The sharp edges, slightly over-smoothed by Xu and Jia [11] and Pan et al. [59], have been reconstructed accurately using our proposed method. Its superior performance benefits from the hybrid blur kernel constraints and edge-preserving TGV${}^{2}$ regularizer.

**Figure 11:**
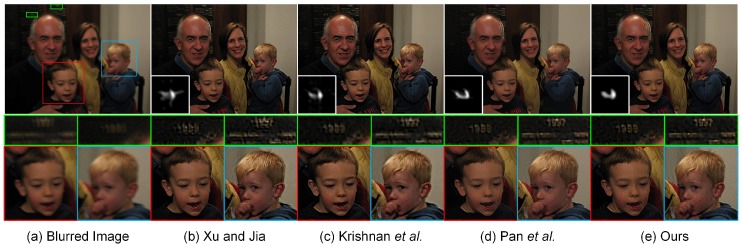
Restoration of a blurred image with a blur kernel of the size 27×27 (top) and the corresponding local magnification views (middle and bottom). From left to right: (**a**) input blurred images, deblurred versions generated by (**b**) Xu and Jia [11], (**c**) Krishnan et al. [9], (**d**) Pan et al. [59] and (**e**) our proposed method, respectively.

**Figure 12:**
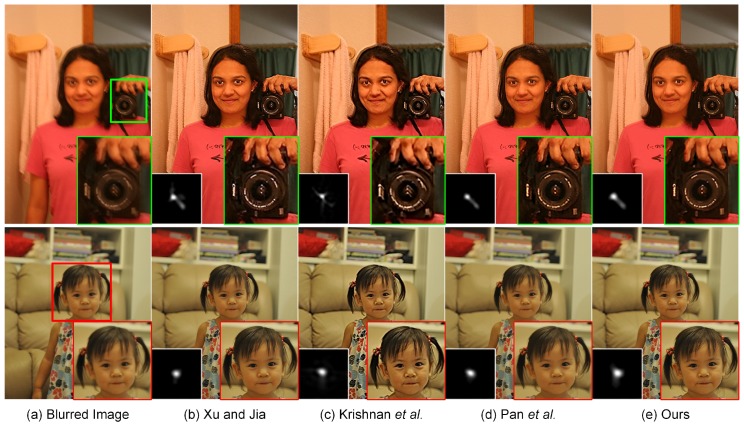
Blind deconvolution of two realistic human images. From left to right: (**a**) input blurred images, deblurred versions generated by (**b**) Xu and Jia [11], (**c**) Krishnan et al. [9], (**d**) Pan et al. [59] and (**e**) our proposed method, respectively. The sizes of the estimated blur kernels from top to bottom are 25×25 and 23×23, respectively.

**Figure 13:**
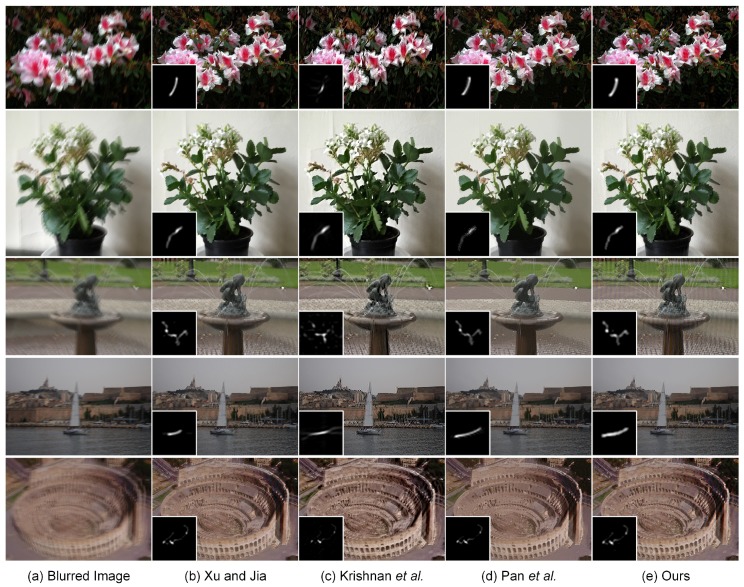
Blind deconvolution of five different realistic natural images. From left to right: (**a**) input blurred images, deblurred versions generated by (**b**) Xu and Jia [11], (**c**) Krishnan et al. [9], (**d**) Pan et al. [59] and (**e**) our proposed method, respectively. The sizes of the estimated blur kernels from top to bottom are 35×35, 55×55, 41×41, 35×35 and 55×55, respectively (the images are best viewed in full-screen mode).

The excellent deblurring performance of our proposed method could also be visually found in Figure 11. As shown in the local magnification views, the inaccurate estimation of the blur kernel for Xu and Jia [11] causes the degraded visual quality with a significant loss of fine details. Krishnan et al. [9] is able to guarantee the quality of blur kernel estimation in this case. However, the final deblurring result tends to be unsatisfactory because the restored “number” could not be preserved correctly, shown in the local magnification views. The main geometrical structures and fine details in the recovered images are preserved by Pan et al. [59] and our proposed method. The ringing-like artifacts could be visually found near the ear region in the deblurring result by Pan et al. [59]. Our proposed method is able to overcome this limitation, but still generates slight ringing-like artifacts in the jaw region. The reason may be that our non-blind deconvolution method summarized in Algorithm 3 is performed with a constant regularization parameter *λ*. To further enhance the image quality, the regularization parameter should be selected spatially variant to suppress the ringing-like artifacts. More realistic deblurring results on human images are visually displayed in Figure 12. It could be observed that our proposed method yields deblurring results that are visually comparable with the current state-of-the-art methods. Figure 13 illustrates the realistic deblurring results on five different natural images. The sizes of the estimated blur kernels from top to bottom are 35×35, 55×55, 41×41, 35×35 and 55×55, respectively. Since these realistic images contain sufficient textures and geometrical structures, all competing methods have the capacity of accurately estimating the blur kernels in these cases. Therefore, our experimental results are visually comparable to others. The quality of the deblurred images could be correspondingly guaranteed in practical applications.

## 5. Conclusions and Future Work

The major contributions of this work are mainly two-fold. First, a hybrid regularization method was developed to robustly estimate the blur kernel by incorporating both the $L_{1}$-norm of kernel intensity and the squared $L_{2}$-norm of the intensity derivative. The underlying assumption behind the proposed method was that the blur kernel was not only spatially sparse, but also piecewise smooth within the support of a continuous curve. An alternating direction algorithm was then proposed to effectively solve the resulting problem of blur kernel estimation. Second, to guarantee high-quality non-blind deconvolution, the TGV${}^{2}$-regularized variational model with an $L_{1}$-norm data-fidelity term was presented to enhance the final image quality. The resulting optimization problem was then effectively solved using an ADMM-based numerical method. Comprehensive experiments implemented on both synthetic and realistic blurred images have illustrated the effectiveness of the proposed method. Given the recent progress in image deblurring, the proposed deblurring framework has several potential limitations in its current version. To further improve the blind deblurring performance, there is a huge potential to extend our future work along the following directions:

- The constant parameters (i.e., $\eta_{1}$ and $\eta_{2}$) for both the $L_{1}$-norm of kernel intensity and the squared $L_{2}$-norm of intensity derivative in (3) are manually selected in our current work. Essentially, it is necessary to automatically and adaptively select the parameters according to the statistical properties of the blur kernel. For instance, if the blur kernel can be better sparsely represented in the spatial domain, $\eta_{1}$ should be larger; whereas $\eta_{2}$ plays a more important role if the blur kernel has a significant piecewise smooth structure. In our future work, an automatic estimation method should be developed to adaptively select the weighting parameters $\eta_{1}$ and $\eta_{2}$ in (3) to enhance the accuracy of blur kernel estimation.
- The single-image blind deblurring method proposed in this work is performed based on a common assumption that the blur kernel is uniform (i.e., spatially invariant) across the image plane. Recent work in the literature [2,60,61,62,63,64,65] has illustrated that the uniform simple assumption does not always hold in practice. To further enhance image quality, the assumption of the non-uniform (i.e., spatially variant) blur kernel has gained increasing attention in modern imaging sciences. In our opinion, the proposed hybrid regularized blur kernel estimation method discussed in Section 2 can be naturally extended to the case of non-uniform deblurring in future work.

As discussed beforehand, our proposed method suffers from some potential limitations (i.e., constant weighting parameters and uniform blurring assumption). Numerous experiments implemented on both synthetic and realistic blurred images have demonstrated its satisfactory deblurring performance. Therefore, it is still worthy of consideration since it could guarantee reliable performance compared to current state-of-the-art uniform blind deblurring methods. Recent research [9,41,59] has indirectly shown that our proposed method could be easily extended to the case of the non-uniform scenario. We believe there is a great potential for restoring blurred images using the proposed method in practical applications. 

## Figures and Tables

**Table 1:** Sum of squared differences (SSD)/CPU computational time (unit: seconds) of different blind deblurring methods on one synthetic test image from [4].

Methods	Ker01	Ker02	Ker03	Ker04	Ker05	Ker06	Ker07	Ker08
	Im02							
Fergus et al. [7]	156.1/449.6	181.8/403.7	44.28/409.3	88.89/311.0	69.13/350.5	44.92/377.8	120.6/407.8	77.30/466.8
Xu and Jia [11]	44.45/1.264	82.76/1.263	76.92/1.061	38.64/1.263	138.2/1.248	67.12/1.279	231.6/1.280	81.19/1.264
Cho and Lee [12]	75.82/1.159	103.2/1.163	74.06/1.021	112.1/1.250	70.55/1.140	41.28/1.133	75.42/1.236	130.6/1.245
Pan and Su [13]	42.11/1.474	102.2/1.564	56.28/1.328	132.5/1.559	46.36/1.428	37.04/1.452	99.73/1.673	57.67/1.572
Levin et al. [58]	85.76/115.9	121.8/138.7	37.92/68.14	73.36/299.1	102.7/94.09	83.74/83.95	106.9/185.6	57.93/179.0
Ours	35.20/2.041	74.64/2.008	36.85/1.823	95.50/2.159	34.26/1.884	34.20/2.052	73.17/2.136	42.25/2.128
	**Im04**							
Fergus et al. [7]	99.55/436.2	162.4/389.7	51.64/418.6	72.67/299.2	47.70/338.7	45.65/374.6	90.47/388.4	65.20/472.4
Xu and Jia [11]	38.43/1.294	93.93/1.248	70.88/1.092	195.7/1.280	60.74/1.263	36.03/1.123	125.3/1.264	69.44/1.263
Cho and Lee [12]	112.0/1.243	113.5/1.130	69.76/1.046	135.2/1.269	116.0/1.223	43.65/1.086	269.0/1.238	123.1/1.242
Pan and Su [13]	45.42/1.494	131.4/1.560	54.48/1.325	127.5/1.572	55.22/1.433	36.64/1.457	125.5/1.579	62.00/1.570
Levin et al. [58]	88.21/108.5	123.3/135.6	39.84/79.05	122.4/284.9	63.71/91.66	63.30/79.67	114.0/169.6	57.70/168.3
Ours	36.43/1.995	93.36/1.970	36.58/1.895	90.51/2.129	39.91/1.867	35.20/1.923	88.96/2.171	45.18/2.163
